# Prevalence and types of lower limb conditions in Nepal

**DOI:** 10.7189/jogh.16.04098

**Published:** 2026-04-30

**Authors:** Lucy Gates, Amos R Channon, Alex Dickinson, Basu D Pandey, Abhinav Vaidya, Binit Vaidya, Yeti Raj Niraula, Rabindra Baskota, Shweta Nakarmi, Cheryl Metcalf, Kate Ward, Alan Silman, Anthony Woolf, Mahesh C Puri

**Affiliations:** 1 School of Healthcare Enterprise & Innovation, University of Southampton, Southampton, UK; 2 Exceed Research Network, Lisburn, UK; 3 Centre for Global Health and Policy, University of Southampton, Southampton, UK; 4 School of Engineering, University of Southampton, Southampton, UK; 5 DEJIMA Infectious Disease Research Alliance (DIDA), Nagasaki University, Japan; 6 Department of Community Medicine, Kathmandu Medical College Public Limited, Kathmandu, Nepal; 7 National Center for Rheumatic Diseases, Kathmandu, Nepal; 8 Technical Unit, Rehabilitation, Humanity and Inclusion, Syria Mission, Raqqa City, Nepal; 9 Leprosy Control and Disability Management Section, Ministry of Health and Population, Nepal; 10 Department of Rheumatology, National Center for Rheumatic Diseases, Kathmandu, Nepal; 11 MRC Lifecourse Epidemiology Centre, University of Southampton, Southampton, UK; 12 Nuffield Department of Orthopaedics, Rheumatology and Musculoskeletal Sciences, University of Oxford, Oxford, UK; 13 Clinical and Molecular Osteoporosis Unit, Department of Clinical Sciences Malmö, Lund University, Malmö, Sweden; 14 Center for Research on Environment Health and Population Activities (CREHPA), Kathmandu, Nepal

## Abstract

**Background:**

Lower limb conditions (LLCs) are a major cause of pain, disability, and loss of livelihood globally, yet their prevalence and functional impacts in low- and middle-income countries, including Nepal, remain poorly described.

**Methods:**

We conducted a cross-sectional, community-based survey in three Nepali districts representing plains, hills, and mountains. Of 2525 screened households, the first 500 adults with self-selected LLCs were interviewed using structured questionnaires adapted from validated tools (Global Alliance for Musculoskeletal Health, COPCORD, Washington Group, WHODAS 2.0). Descriptive statistics summarised condition type, pain location, activity limitations, employment impact, and comorbidities. χ^2^ tests assessed regional differences in condition types.

**Results:**

Of 2525 households, 671 (26.6%, 95% CI = 24.9–28.3) reported a member with an LLC; 11.2% (95% CI = 10.5–12.0) of adults were affected. Among 500 participants surveyed (mean age 57 years; 65% female), pain/discomfort was most common (97%), mainly in the knee (74%) and foot/ankle (48%). Across participants, 628 LLCs were reported; conditions included injury/trauma (19%), deformity (7%), wounds (1%), and amputation (<1%). Prevalence varied by district. Functional limitations were substantial: 82% with pain and all with amputation reported severe activity restriction. Ten percent were unemployed, mostly due to health, and >70% of those doing household work had left other jobs because of their LLC.

**Conclusions:**

This study offers preliminary, population-based estimates of person-reported LLCs across three ecological zones in Nepal. Musculoskeletal pain was most common, often multi-site and substantially limiting daily activities and employment. While findings highlight the impact of LLCs on well-being and livelihoods, they remain exploratory due to limited geographic scope and self-reporting. Larger, nationally representative studies are needed to confirm these results, differentiate chronic from transient pain, and guide rehabilitation and prevention strategies.

Understanding the burden and pattern of health conditions is vital for health systems to plan services and allocate resources effectively. In many low- and middle-income countries (LMICs), data are limited and often derived from Global Burden of Disease (GBD) estimates, which rely on broad categories of conditions [1]. This can obscure the lived realities of conditions that are not captured; those that profoundly affect mobility, independence, and economic participation, such as those involving the lower limb.

Lower limb conditions (LLCs) can include congenital abnormalities, neurological and vascular disorders, musculoskeletal pathologies, injuries, and amputations. Although encompassing a diverse range of conditions, the LLC concept is useful as it captures the overall burden of conditions which are known to lead to pain, disability, and reduced quality of life, with consequences that extend to households and communities [2–4]. In LMICs, the burden of LLCs is often overlooked, as health systems remain focused on communicable diseases and selected non-communicable diseases. Evidence from South Asia suggests that musculoskeletal pain and injuries are widespread, frequently underdiagnosed, and poorly managed, leading to loss of productivity, social exclusion, and long-term disability [5,6].

Nepal faces multiple challenges relevant to LLCs, including rugged terrain, limited access to health services, high rates of road traffic injury [7], and a growing burden of type 2 diabetes [8]. Over one-third of Nepals population lives in rural areas with restricted access to rehabilitation and specialist care [9]. Limited evidence suggests approximately 2.35 million people in Nepal live with musculoskeletal conditions, with a prevalence of 14.8% and an unmet treatment need of 60%. Over one-third of these are estimated to relate to the lower limb [10]. A recent rural community-based study using the COPCORD tool estimated the prevalence of musculoskeletal pain/swelling (past and current) at 26.81% (95% CI = 24.9–28.7) [5]. Since LLCs include a broader range of conditions, this suggests an even larger portion of the population may be affected. We do not know the distribution of these conditions across Nepal or how they affect households’ economic security, employment, or access to health care. Given the importance of mobility for livelihoods, especially in agricultural and manual labour contexts, the impact of LLCs on Universal Health Coverage (UHC) and progress towards the Sustainable Development Goals (SDGs, 3 and 10) is likely to be substantial but remains poorly quantified.

A significant challenge in estimating LLC prevalence in LMICs is the difficulty in collecting accurate data in populations with limited access to diagnostics, health records, and registries. Capturing all symptoms affecting daily living and mobility is essential. Thus, a holistic self-report approach to LLC criteria may more effectively estimate their scale and impact. Capturing the prevalence and types of LLCs, as well as their impact on function and livelihoods, is a critical first step to informing health policy, workforce training, and service development. However, there is currently no surveillance system or institution in Nepal dedicated to monitoring these conditions. This study therefore sought to provide preliminary estimates of the prevalence and types of LLCs across three ecological zones of Nepal (plains, hills, and mountains), examining their distribution by type and by key personal and ecological factors, as well as their effect on daily functioning.

## METHODS

Findings were reported using STROBE guidelines for cross-sectional studies (Table S1 in the **Online Supplementary Document**).

### Study design and setting

We conducted a community-based, cross-sectional survey across three districts of Nepal – Dang (plains/terai), Lamjung (hill), and Dolakha (mountain) – chosen to represent the country’s three ecological zones.

### Study population

A total of 2525 households were screened between July and August 2021. Adults aged ≥18 years reporting a current lower limb condition, defined as a current (new or longstanding) problem with their leg or foot, were eligible. The first 500 adults after screening of 2525 households from three districts were surveyed (Dang, n = 200; Dolakha, n = 100; and Lamjung, n = 200).

### Sampling strategy

A three-staged cluster sampling approach was used. Within each district, clusters (wards of municipalities) were selected using probability proportional to size, stratified by rural and urban settings. Within ten clusters, households were systematically approached for screening. This strategy was designed to balance feasibility with geographic diversity, and the smaller Dolakha sample reflects greater access challenges.

### Case definition of LLCs

Lower Limb Conditions were defined broadly to include pain/discomfort, deformity, amputation, wounds, and injuries/trauma affecting the hip, thigh, knee, calf, ankle, or foot (Table S2 in the **Online Supplementary Document**). The definition was adapted from the Global Alliance for Musculoskeletal Health survey module [11], the Community Oriented Program for Control of Rheumatic Diseases (COPCORD) tools [12] the Washington Group questions [13] and the WHO Disability Assessment Schedule [14], in consultation with an advisory committee (Table S3 in the **Online Supplementary Document**). This inclusive approach was intended to capture the spectrum of LLCs in a context where diagnostic services are limited.

Pain and/or discomfort was defined as any pain or discomfort affecting muscles or joints in the lower limb in the past month, reported by the participants by indicating from 26 reference pain locations provided on a lower body manikin (Figure S1 in the **Online Supplementary Document**). ‘Amputation’ was based on any previous amputation at the leg or foot. ‘Lower limb deformity’ was either congenital or acquired. ‘Injury or trauma’ was any that had been suffered that had left a lasting effect on the lower limb, and ‘wounds’ was an open sore on the leg or foot in the past month that had taken over 2 weeks to heal. Participants could report more than one condition and the location of condition.

### Questionnaire and data collection

The questionnaire collected information on condition type and location, comorbidities, treatment-seeking behaviours, functional impact, and socioeconomic consequences. Questions on daily activity limitations were adapted from WHODAS 2.0. Participants rated the extent to which each reported condition affected their ability to carry out activities of daily living.

Questionnaires were first developed in English and translated into Nepali, pre-tested and programmed into the data collection software KoboToolbox (Kobo Inc., Toronto, Canada). Eleven research assistants were trained by a UK-qualified Podiatrist (LG) in the recognition and understanding of lower limb conditions. They obtained written informed consent and conducted survey interviews in person in a private space in participants’ homes using electronic tablets. Participants who were illiterate provided thumbprints to confirm consent.

### Data analysis

All analysis was completed in Stata version 19.0 (Stata Corp, College Station, Texas, USA). Prior to analysis, data distributions were checked for inconsistencies, outliers, and missing information. Household and individual prevalence estimates were calculated with appropriate denominators and 95% confidence intervals, based on either entire surveyed household population or those only reporting an LLC. Frequencies of condition types were stratified by region, sex, and age group. χ^2^ tests were used to compare prevalence across regions, with *P* < 0.05 considered as statistically significant.

## RESULTS

A total of 2525 households were surveyed and screened for individuals with LLCs. Of these, 671 households (26%, 95% confidence interval (CI) = 24.9–28.3) reported at least one member with an LLC. At the person level, 11.2% (95% CI = 10.5–12.0) of adults screened (aged over 18) were affected.

### Characteristics of participants

Among the 500 participants with an LLC that were surveyed (Dang, n = 200; Lamjung, n = 200; Dolakha, n = 100), the mean age was 57.5 years (SD = 14.8), and 65% were female (**Table 1**).

**Table 1:** Description of households screened in the overall survey

Characteristics*	n = 2525
Age of household member in years, mean (SD)	32 (21.6)
Sex of head of the household	
*Female*	1529 (60.6)
*Male*	996 (39.4)
District name	
*Dang*	1006 (39.8)
*Dolakha*	500 (19.8)
*Lamjung*	1019 (40.4)
Presence of at least one LLC in household	
*Present*	671 (26.6)
Type of LLC	
*Pain or discomfort*	486 (19.2)
*Injury/trauma*	95 (3.8)
*Deformity*	36 (1.4)
*Wound*	7 (0.3)
*Amputation*	4 (0.2)

LLC – lower limb conditions, SD – standard deviation

*Values are presented as n (%) unless specified otherwise.

Between 8% and 19% of participants self-reported osteoarthritis in at least one lower limb joint. Diabetes and neurological conditions were more commonly reported in Dang (plains), while fracture history was more frequent in Dolakha (mountain) (**Table 2**).

**Table 2:** Description of individuals reporting an LLC (n = 500)

Characteristics*	Dang (terai), n = 200	Dolakha (mountain), n = 100	Lamjung (hill), n = 200
Age in years, mean (SD)	55.8 (15.9)	56.2 (13.2)	58.1 (14.3)
Sex of head of the household			
*Female*	128 (64.0)	66 (66.0)	132 (66.0)
*Male*	72 (36.0)	34 (34.0)	68 (34.0)
Medical conditions			
*Osteoarthritis*	38 (19.0)	8 (8.0)	30 (15.0)
*Fractures*	7 (3.5)	14 (14.0)	2 (1.0)
*Diabetes*	22 (11.0)	8 (8.0)	20 (10.0)
*Neurological conditions*†	12 (6.0)	6 (6.0)	11 (5.5)

LLC – lower limb conditions, SD – standard deviation

*Values are presented as n (%) unless specified otherwise.

†Including stroke, Parkinson, cerebral palsy, spinal cord injury, multiple sclerosis and other neurological disease.

### Types of LLCs

**Figure 1:**
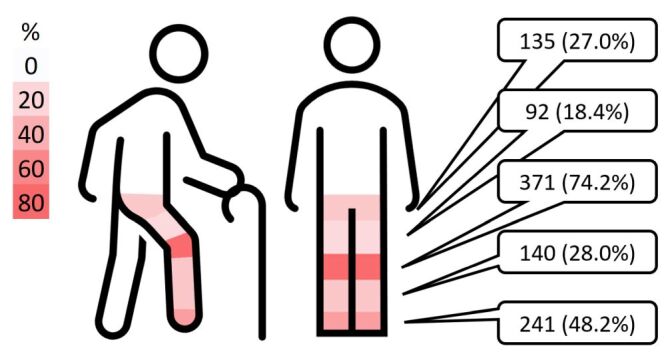
Prevalence of self-reported pain or discomfort in the hip, thigh, knee, calf, ankle or foot, reported as n (%).

**Figure 2:**
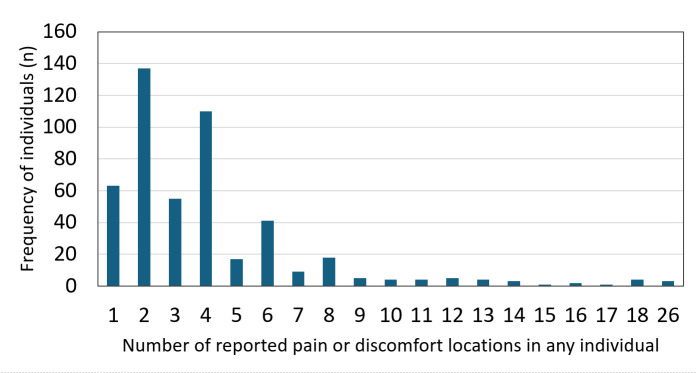
Frequency of individuals reporting one or more locations for lower limb pain or discomfort.

In total, 628 LLCs were reported by 500 participants, reflecting the fact that some individuals experienced more than one condition. Pain and discomfort were the most common, reported by 486 of the 500 participants surveyed (97.2%, 95% CI = 95.4–98.5) (**Table 3**). Among these, the knee was the most frequently affected site (n/N = 371/500, 74.2%; 95% CI = 70.2–78.0), followed by the foot/ankle (n/N = 241/500, 48.2%; 95% CI = 43.9–52.6) (**Figure 1**). A high proportion reported pain in multiple locations, with three individuals reporting pain in all 26 sites assessed (**Figure 2**).

**Table 3:** Percentage of individuals reporting different conditions by background characteristics

Characteristics	Total	Pain or discomfort, n (%)	Injury/trauma, n (%)	Deformity, n (%)	Wound, n (%)	Amputation, n (%)
Sex						
*Male*	174	162 (93.1)	29 (16.7)	21 (12.1)	6 (3.4)	4 (2.3)
*Female*	326	324 (99.4)	66 (20.2)	15 (4.6)	1 (0.3)	0 (0.0)
Age						
*18–39*	67	63 (94.0)	14 (20.9)	11 (16.4)	1 (1.5)	2 (3.0)
*40–54*	141	138 (97.9)	33 (23.4)	6 (4.3)	1 (0.7)	1 (0.7)
*55–69*	187	182 (97.3)	31 (16.6)	10 (0.5)	3 (1.6)	1 (0.5)
*70+*	105	103 (98.1)	17 (16.2)	9 (8.6)	2 (1.9)	0 (0.0)

Other conditions were less common: 95 participants (19.0%, 95% CI = 15.7–22.7) reported lower limb injury/trauma, 36 (7.2%, 95% CI = 5.1–9.8) reported deformity, seven (1.4%, 95% CI = 0.6–2.9) reported a wound, and four (0.8%, 95% CI = 0.2–2.0) reported an amputation. These small subgroups should be interpreted with caution, as prevalence estimates are unstable due to low case numbers.

More females reported lower limb pain or discomfort (99.4% *vs.* 93.1%) and injury/trauma (20.2% *vs.* 16.7%) than males. Conversely more males reported lower limb deformity (12.1% *vs.* 4.6%) and wounds (3.4% *vs.* 0.3%) (**Table 3**).

### Regional patterns

The proportion of participants reporting pain/discomfort and deformity was relatively similar across the three districts. Injury/trauma was significantly more common in Dolakha (26%, 95% CI = 17.7–35.7) compared to Dang (24%, 95% CI = 18.3–30.5) and Lamjung (10.5%, 95% CI = 6.6–15.6) (χ^2^ test, *P* < 0.001). Wounds were most frequently observed in Dang, though absolute numbers were very small (Table S5 in the **Online Supplementary Document**).

### Functional impact

All participants with amputation (0.8%, 95% CI = 0.3–2.0) reported that their condition severely or extremely affected their ability to carry out activities of daily living (ADLs). Eighty two percent (95% CI = 78.4–85.3) of those with pain/discomfort, (71%, 95% CI = 35.9–91.8) with a wound, (59%, 95% = 48.9–68.3) with injury/trauma, and (56%, 95% CI = 39.6 – 70.5) with deformity reported their condition severely or extremely affected their ability to carry out ADLs. Only in cases of deformity (22%, 95% CI = 11.7 – 38.1) and injury/trauma (2.1%, 95% CI = 0.6–7.4) did any participants report their condition did not affect ADLs.

### Employment impact

Ten percent (95% CI = 7.4–12.6) of participants reported being unemployed, with 96% (95% CI = 90.6–100) attributing this to a health condition or disability. A majority of these unemployed individuals were aged over 60 years, so are also near or older than the official retirement age (with only a further seven individuals reporting that they were retired). A further 41.4% (95% CI = 37.1 – 45.7) reported household chores as their main occupation, with the 90% (95% CI = 85.9–94.1) of these undertaken in their own homes. This was at all ages, potentially indicating the limited ability of LLC sufferers to obtain an occupation. Over 70% of those reporting household chores stated that they had given up their work due to their LLC, indicating that the chores were not a choice of occupation for many.

## DISCUSSION

This community-based survey provides preliminary estimates of the prevalence and types of lower limb conditions (LLCs) in three ecological zones of Nepal, alongside their reported functional and employment impacts. More than one in ten adults adults screened had an LLC. Pain and discomfort were the most common presentation, consistent with studies from India and rural Nepal reporting musculoskeletal pain prevalence above 20–25% [5,15]. Pain and injury were slightly more common in women, whereas deformities and wounds were more frequent in men. These differences may reflect factors such as greater occupational exposure to physically demanding and hazardous work, cultural norms leading men to delay care, inadequate protective footwear, and higher prevalence of risk factors like smoking, alcohol use, and diabetes among men [16], which can impair healing and bone health. Knee and foot/ankle involvement were especially common, aligning with evidence that these sites carry a high burden of musculoskeletal disability globally [1]. This high prevalence highlights the significant burden of musculoskeletal issues in this population. Musculoskeletal pain, especially joint and back pain, is the most common type of chronic pain and is the main contributor to disability worldwide [17]. According to the WHO, 20–33% of the world’s population has some form of chronic musculoskeletal pain, translating to 1.75 billion people globally. Data from the Global Burden of Disease study showed the prevalence of other musculoskeletal disorders including a wide range of joint, ligament, tendon, or muscle problems that cause regional or generalised pain was 8.4% (95% uncertainty interval (UI) = 8.1%–8.6%) [18].

The functional and economic impacts identified in this study were considerable. Over 80% of participants with musculoskeletal pain described severe or extreme limitations in daily activities, and a substantial proportion reported withdrawal from employment, reflective of findings from rural India [19], where musculoskeletal pain is a substantial cause of work loss and household economic strain.

Some regional variation was observed, with injury and trauma more common in Dolakha. This may reflect differences in terrain, occupational exposures and access to acute care. Dolakha is a mountain district with more rugged terrain, where geographical features and topography may heighten the risks of accidents and injuries, however the sample size for Dolakha was smaller, and these findings require confirmation in larger-scale studies. Additionally, socio-economic factors such as infrastructure development, road conditions, and access to health care services differ between regions. Cultural and behavioural factors may also be related to risk-taking behaviours, safety practices, and awareness of injury prevention measures might also vary between regions, influencing the reporting rates of injuries and trauma. The very small numbers of participants with deformity, wounds, or amputation are useful indicators of proportion, but are insufficient to drive population-level policy without further evidence.

These differences between regions emphasise the varying patterns of health challenges faced within Nepal, emphasising the importance of tailored health care interventions to address specific needs effectively. As per the recent Department of Health Services of Nepal annual report [20], progress is being made across disability-inclusive health, rehabilitation, assistive technology, and injury prevention. For example, Nepal has seen the piloting of a disability management and rehabilitation training package for primary health care providers, plus the development of the national standard on assistive technology and priority assistive product list. Furthermore, the Systematic Assessment of Rehabilitation Situation report (STARS) [21] was finalised and the Rapid Assistive Technology Assessment (rATA) [22,23] was conducted in coordination with the Nepal Health Research Council. Preliminary data has been collected to evaluate the rehabilitation workforce using WHO-standardised tools.

Our results highlight the impact of various LLCs on individuals' ability to perform activities of daily living and undertake their chosen occupation in Nepal. Notably, all participants with amputation reported that their condition severely or extremely affected their daily activities, indicating the significant functional limitations faced by this subgroup. Furthermore, the high percentages of individuals reporting severe or extreme limitations in carrying out activities of daily living across other LLCs are notable. These findings highlight the broad-ranging impact of LLCs on individuals' functional abilities and quality of life, regardless of the specific nature of their condition.

### Strengths and limitations

This is among the first community-based studies in Nepal to estimate the prevalence of a broad range of person-reported LLCs. Strengths include a relatively large population that was screened (2525 households), representation across three ecological zones, and use of adapted, validated instruments. However, several limitations must be acknowledged. Only three districts were included. While these areas provide diverse geographic and social contexts, they do not capture the full heterogeneity of Nepal’s 77 districts and seven provinces, but they offer an important first step in describing the burden of LLCs and their functional and economic consequences.

Reliance on self-report is subject to misclassification and under or over-reporting/estimation: pain and discomfort may have been interpreted differently across settings, and we could not distinguish chronic, activity-limiting pain from discomfort. However, this was judged a necessary compromise given that there is limited access to diagnostic health care in many regions of Nepal. Social desirability bias may also be present, where respondents may have provided answers to questions in such a way as to present themselves in socially acceptable terms. Five days intensive interviewer training was conducted, but inter-rater reliability was not formally assessed. Finally, while WHODAS items were included, full scoring was not feasible, so functional impacts are described descriptively rather than quantitatively.

### Implications for research and practice

The findings highlight the potential burden of LLCs in Nepal and their significant functional consequences. These results are hypothesis-generating and larger, nationally representative studies are needed to confirm prevalence estimates, characterise condition subgroups, and identify high-risk populations with larger sample size and coverage. Future research should also examine access to care, costs of assistive technology, and barriers to rehabilitation in greater depth.

## CONCLUSIONS

This study provides preliminary, community-based evidence of the prevalence and types of person-reported LLCs in in three ecological zones of Nepal. Pain was common, often affecting multiple sites and substantially limiting daily activities and employment. Small numbers of participants reported deformities, wounds, and amputations. While limited by its geographic scope we covered Nepal’s three key ecological zones to make results as generalisable as possible. Whilst we relied on self-report, the study demonstrates the value of community-based surveys for capturing conditions that are currently underrepresented in existing surveillance systems. The findings raise the profile of this neglected widespread area and have set the context where a larger-scale investigation can be conducted.

### Recommendations

Based on our findings, we provide a number of recommendations.

#### Research priorities

We recommend that larger-scale, nationally representative studies be conducted to better estimate the prevalence and distribution of LLCs across Nepal’s diverse ecological and cultural settings, using additional standardised diagnostic and functional measures wherever possible.

#### Service needs

We recommend strengthening rehabilitation and assistive device provision in rural areas, particularly for musculoskeletal pain and mobility limitations. We also recommend that high-level support be provided to enable people with disabilities to continue working, or that bridging loans be offered where needed to allow individuals to recommence work after recovery, as this would benefit both individuals and the wider economy.

#### Policy relevance

We recommend using these findings to raise awareness of LLCs as an under-recognised burden within UHC planning. However, we advise that any national-scale system redesign be based on more comprehensive evidence than is currently available.

## Additional material


Online Supplementary Document
