# Supplementary material for: Prevalence and types of lower limb conditions in Nepal
Source: J Glob Health. 2026 Apr 30;16:04098. doi: 10.7189/jogh.16.04098 (PMC13136662; doi:10.7189/jogh.16.04098)
Supplement: Online Supplementary Document [file jogh-16-04098-s001.pdf]

Supplement to: Gates L, Channon A, Dickinson A, Pandey BD, Vaidya A, Vaidya B, Niraula Y, Baskota R, Nakarmi S, Metcalf C, Ward K, Silman A, Woolf A, Puri MC. Prevalence and types of lower limb conditions in Nepal. J Glob Health. 2026;16:04098.

Table S1. STROBE Statement—checklist of items that should be included in reports of observational studies

	Item No.	Recommendation	Page No.
**Title and abstract**	1	(*a*) Indicate the study's design with a commonly used term in the title or the abstract	1
		(*b*) Provide in the abstract an informative and balanced summary of what was done and what was found	2
**Introduction**			
Background/rationale	2	Explain the scientific background and rationale for the investigation being reported	2-4
Objectives	3	State specific objectives, including any prespecified hypotheses	3-4
**Methods**			
Study design	4	Present key elements of study design early in the paper	4
Setting	5	Describe the setting, locations, and relevant dates, including periods of recruitment, exposure, follow-up, and data collection	4
Participants	6	Give the eligibility criteria, and the sources and methods of selection of participants	4
Variables	7	Clearly define all outcomes, exposures, predictors, potential confounders, and effect modifiers. Give diagnostic criteria, if applicable	4-5
Data sources/ measurement	8*	For each variable of interest, give sources of data and details of methods of assessment (measurement). Describe comparability of assessment methods if there is more than one group	4-5
Bias	9	Describe any efforts to address potential sources of bias	5, 9 and 10
Study size	10	Explain how the study size was arrived at	4

Quantitative variables	11	Explain how quantitative variables were handled in the analyses. If applicable, describe which groupings were chosen and why	5-6
Statistical methods	12	(*a*) Describe all statistical methods, including those used to control for confounding	5-6
		(*b*) Describe any methods used to examine subgroups and interactions	
		(*c*) Explain how missing data were addressed	5-6
		(*d*) *Cross-sectional study*—If applicable, describe analytical methods taking account of sampling strategy	5-6
		(*e*) Describe any sensitivity analyses	
Participants	13*	(a) Report numbers of individuals at each stage of study—eg numbers potentially eligible, examined for eligibility, confirmed eligible, included in the study, completing follow-up, and analysed	6
		(b) Give reasons for non-participation at each stage	6
		(c) Consider use of a flow diagram	
Descriptive data	14*	(a) Give characteristics of study participants (eg demographic, clinical, social) and information on exposures and potential confounders	6 and Table 1 and 2
		(b) Indicate number of participants with missing data for each variable of interest	
Outcome data	15*	*Cross-sectional study*—Report numbers of outcome events or summary measures	6-7
Main results	16	(*a*) Give unadjusted estimates and, if applicable, confounder-adjusted estimates and their precision (eg, 95% confidence interval). Make clear which confounders were adjusted for and why they were included	6-7, Table 3 and Supplementary material (S5 and 6)
		(*b*) Report category boundaries when continuous variables were categorized	Table 3 and Supplementary material (S5 and 6)
		(*c*) If relevant, consider translating estimates of relative risk into absolute risk for a meaningful time period	

Other analyses	17	Report other analyses done—eg analyses of subgroups and interactions, and sensitivity analyses	
Key results	18	Summarise key results with reference to study objectives	7-9
Limitations	19	Discuss limitations of the study, taking into account sources of potential bias or imprecision. Discuss both direction and magnitude of any potential bias	9-10
Interpretation	20	Give a cautious overall interpretation of results considering objectives, limitations, multiplicity of analyses, results from similar studies, and other relevant evidence	9-10
Generalisability	21	Discuss the generalisability (external validity) of the study results	9-10
**Other information**			
Funding	22	Give the source of funding and the role of the funders for the present study and, if applicable, for the original study on which the present article is based	12

*Give information separately for cases and controls in case-control studies and, if applicable, for exposed and unexposed groups in cohort and cross-sectional studies.

Note: An Explanation and Elaboration article discusses each checklist item and gives methodological background and published examples of transparent reporting. The STROBE checklist is best used in conjunction with this article (freely available on the Web sites of PLoS Medicine at http://www.plosmedicine.org/, Annals of Internal Medicine at http://www.annals.org/, and Epidemiology at http://www.epidem.com/). Information on the STROBE Initiative is available at www.strobe-statement.org.

Table S2. Definitions for each condition

Condition	Definition	Recall period
Pain and/or discomfort (musculoskeletal condition)	Any pain or discomfort affecting your muscles or joints in your lower limb (hip, thigh, knee, calf, ankle or foot)<br>→Followed by indicating from 26 reference pain locations provided on a lower body manikin (appendix 3)	Past month
Amputation	Previous amputation at the leg or foot<br>→Followed by a choice of multiple locations to the indicated region of amputation	Ever
Deformity	Any deformity you may have at your leg or foot, that you were born with or acquired at any age<br>→Followed by indicating from 26 reference pain locations provided on a lower body manikin	Ever
Injury or trauma	An injury or trauma to your lower limb that has left a lasting effect on your leg<br>→Followed by indicating from 26 reference pain locations provided on a lower body manikin	Past month
Wound	An open sore on your leg or foot that has taken over 2 weeks to heal – (This does not include a blister, verruca, bruise or rash)<br>→Followed by a choice of multiple locations to indicate region of amputation	Past month
Other conditions	Any altered sensation (numbness/tingling, muscle weakness) in your legs or feet	Past month

	→Followed by a choice of multiple locations to indicate region of amputation	

Table S3. Lower Limb conditions in Nepal- Advisory Group Members

Member	Role and affiliation
Professor Amos Channon	Co-lead. Professor in Demography, University of Southampton
Dr Lucy Gates	Co-lead. Senior Lecturer and Global Health Researcher, University of Southampton
Dr Mahesh Puri	Co-Investigator. Centre for Research on Environment, Health and Population Activities (CREHPA), Nepal
Dr Basu Dev Pandey	Co-chair. Joint secretary at Ministry of Health & Population, former Director of the Epidemiology and Disease Control Division, Ministry of Health and Population
Mr Yeti Raj Niraula	Senior Prosthetist and Orthotist at Humanity & Inclusion (iNGO), Nepal
Dr. Binit Vaidya	Rheumatologist, National Center for Rheumatic Diseases, Kathmandu, Nepal
Professor Abhinav Vaidya	Professor at Kathmandu Medical College, Kathmandu, Nepal
Professor Anthony Woolf	Consultant Rheumatologist and honorary Professor of Rheumatology, University of Exeter Medical School, and Plymouth Peninsula Medical and Dental College
Professor Cheryl Metcalf	Professor of Health Innovation & Technology. Executive Director: MSc Medical Technology, Innovation & Design, University of Southampton
Professor Alan Silman	Epidemiologist and Rheumatologist, University of Oxford
Professor Kate Ward	Professor of Global Musculoskeletal Health, MRC Lifecourse Epidemiology Unit, University of Southampton

Figure S1. Regions to report lower limb pain/discomfort on mannequin

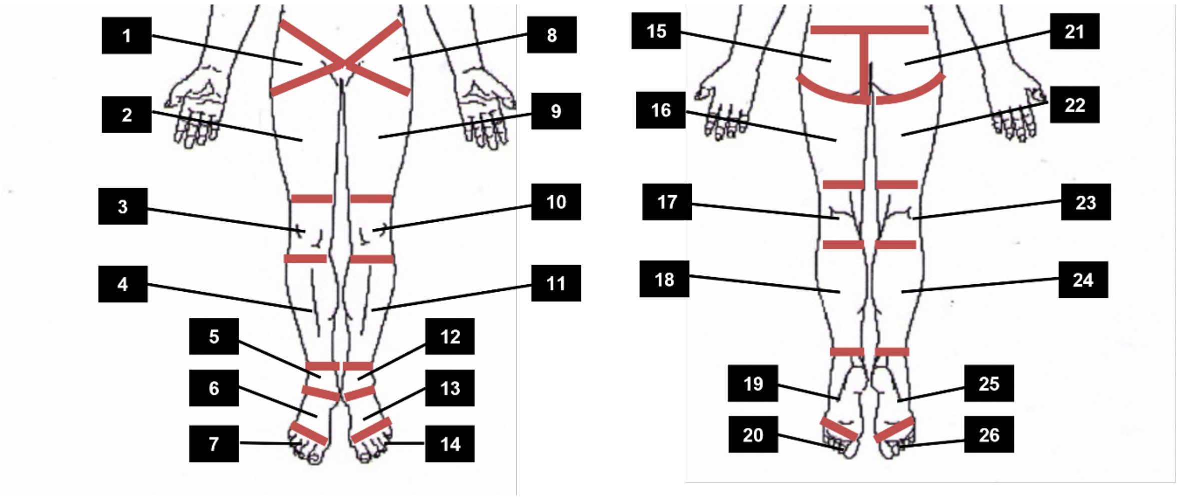

Table S5. Proportion of individuals with each type of LLC (compared to without) by region

	Dang (n=200)	Dolakha (n=100)	Lamjung (n=200)	
Pain or Discomfort, n (% yes)	192 (96.0)	99 (99.0)	195 (97.5)	p=0.314
Injury/Trauma, n (% yes)	48 (24.0)	26 (26.0)	21 (10.5)	p<0.001
Deformity, n (% yes)	14 (7.0)	8 (8.0)	14 (7.0)	p=0.942
Wound, n (% yes)	6 (3.0)	0 (0.0)	1 (0.5)	-
Amputation, n (% yes)	0 (0.0)	3 (3.0)	1 (0.5)	-

*differences tested using chi-squared (χ2). Differences in the proportion of wounds and amputations between countries were not tested due to low counts

Table S6. Proportion of Households with LLC (compared to households without) by region

	Dang (n=1006)	Dolakha (n=500)	Lamjung (n=1,019)	
Not present	789	353	712	
Present	217	147	307	p<0.001

*differences tested using chi-squared (χ2)
